# 1122. Effect of Aluminum Hydroxide/Magnesium Hydroxide/Simethicone and Omeprazole on the Pharmacokinetics of Tebipenem Pivoxil Hydrobromide (TBP-PI-HBr) in Healthy Adult Subjects

**DOI:** 10.1093/ofid/ofab466.1315

**Published:** 2021-12-04

**Authors:** Vipul K Gupta, Gina Patel, Leanne Gasink, Floni Bajraktari, Yang Lei, Akash Jain, Praveen Srivastava, Angela Talley

**Affiliations:** 1 Spero Therapeutics, Inc., Cambridge, Massachusetts; 2 Patel Kwan Consultancy LLC, Madison, Wisconsin; 3 Spero Therapeutics, Cambridge, Massachusetts

## Abstract

**Background:**

Tebipenem pivoxil hydrobromide (TBP-PI-HBr) is an oral prodrug that is converted to tebipenem (TBP), the active moiety being developed for treating complication urinary tract infections. Antacids and proton pump inhibitors are known to change gastric pH after administration, which could affect the absorption of oral medications. This study evaluated the effect of a single dose of aluminum hydroxide/magnesium hydroxide/simethicone and the effect of multiple doses of omeprazole on the PK of TBP, following a single dose of TBP-PI-HBr.

**Methods:**

This was an open-label, 3-period, fixed sequence drug-drug interaction study. On Day 1, Period 1, subjects received a single oral dose of TBP-PI-HBr 600 mg (2 x 300 mg tablets) at Hour 0. On Day 1, Period 2, subjects received a single oral 20 mL dose of aluminum hydroxide 800 mg/magnesium hydroxide 800 mg/simethicone 80 mg suspension per 10 mL (Maalox® Advanced Maximum Strength oral suspension) with a single oral dose of TBP-PI-HBr 600 mg at Hour 0. In Period 3, on Days 1 through 5, subjects received a single oral dose of omeprazole 40 mg (Prilosec®) once daily (QD), at Hour -2. On Day 5, a single oral dose of 600 mg TBP-PI-HBr was administered at Hour 0. Whole blood sampling for TBP PK occurred pre-dose and up to 24 hours post dose. Whole blood samples were assayed for TBP by liquid chromatography-tandem mass spectrometry.

**Results:**

Twenty subject were enrolled and completed the study. Geometric mean ratios for AUC indicated mean TBP exposure (AUC) was approximately 11% lower and mean C_max_ was 22% lower for TBP-PI-HBr combined with aluminum hydroxide/magnesium hydroxide/simethicone vs. TBP-PI-HBr alone (**Figure**). Similarly, geometric mean ratios for AUC indicated mean TBP exposure (AUC) was approximately 11% lower and mean C_max_ was 43% lower for TBP-PI-HBr in combination with omeprazole vs. TBP-PI-HBr alone. Because the PK/PD driver for TBP efficacy is AUC dependent, concomitant administration is not expected to impact the efficacy of oral TBP-PI-HBr.

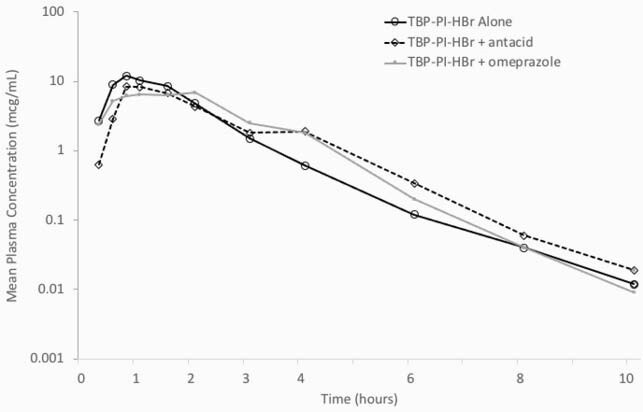

Figure 1. Arithmetic mean plasma TBP concentrations following a 600 mg dose of clinical study drug product (A1 and A2) and registrational drug product (B) – PK population.

**Conclusion:**

Administration of TBP-PI-HBr combined with aluminum hydroxide/magnesium hydroxide/simethicone or omeprazole QD had no meaningful effect on plasma TBP exposure; C_max_ decreased with both agents. Co-administration was generally safe and well tolerated.

**Disclosures:**

**Vipul K. Gupta, Ph.D.**, **Spero Therapeutics** (Employee, Shareholder) **Gina Patel, PhD**, **Spero Therapeutics, Inc.** (Consultant) **Leanne Gasink, MD**, **Spero Therapeutics, Inc.** (Consultant) **Floni Bajraktari, MSc**, **Spero Therapeutics, Inc.** (Employee) **Yang Lei, PhD**, **Spero Therapeutics, Inc.** (Employee) **Akash Jain, PhD**, **Spero Therapeutics, Inc.** (Employee) **Praveen Srivastava, MS, BS**, **Spero Therapeutics, Inc.** (Employee) **Angela Talley, MD**, **Spero Therapeutics, Inc.** (Employee)

